# Genome-wide identification of PEBP gene family in pineapple reveal its potential functions in flowering

**DOI:** 10.3389/fpls.2023.1277436

**Published:** 2023-10-26

**Authors:** Xiaohan Zhang, Yanwei Ouyang, Lei Zhao, Ziqiong Li, Hongna Zhang, Yongzan Wei

**Affiliations:** ^1^ School of Breeding and Multiplication (Sanya Institute of Breeding and Multiplication), Hainan University, Sanya, China; ^2^ Key Laboratory of Biology and Genetic Resources of Tropical Crops, Ministry of Agriculture, Institute of Tropical Bioscience and Biotechnology, Chinese Academy of Tropical Agricultural Sciences, Hainan Institute for Tropical Agricultural Resources, Haikou, China

**Keywords:** pineapple (*Ananas comosus* (L.) Merr.), PEBP, genome-wide, expression profiles, flowering

## Abstract

Phosphatidylethanolamine binding protein (PEBP) plays an important role in regulating flowering time and morphogenesis of plants. However, the identification and functional analysis of *PEBP* gene in pineapple (*AcPEBP*) have not been systematically studied. The pineapple genome contained 11 PEBP family members, which were subsequently classified into three subfamilies (FT-like, TFL-like and MFT-like) based on phylogenetic relationships. The arrangement of these 11 shows an unequal pattern across the six chromosomes of pineapple the pineapple genome. The anticipated outcomes of the promoter cis-acting elements indicate that the *PEBP* gene is subject to regulation by diverse light signals and endogenous hormones such as ethylene. The findings from transcriptome examination and quantitative real-time polymerase chain reaction (qRT-PCR) indicate that FT-like members *AcFT3* and *AcFT4* display a heightened expression level, specifically within the floral structures. The expression of *AcFT3* and *AcFT4* increases sharply and remains at a high level after 4 days of ethylene induction, while the expression of *AcFT7* and *AcMFT1* decreases gradually during the flowering process. Additionally, *AcFT3*, *AcFT4* and *AcFT7* show specific expression in different floral organs of pineapple. These outcomes imply that members belonging to the FT-like subfamily may have a significant impact on the process of bud differentiation and flower development. Through transcriptional activation analysis, it was determined that *AcFT4* possesses transcriptional activation capability and is situated in the nucleus and peripheral cytoplasm. Overexpression of *AcFT4* in *Arabidopsis* resulted in the promotion of early flowering by 6-7 days. The protein interaction prediction network identified potential flower regulators, including CO, AP1, LFY and SOC1, that may interact with PEBP proteins. This study explores flower development in pineapple, thereby serving as a valuable reference for future research endeavors in this domain.

## Introduction

Phosphatidylethanolamine-binding proteins (PEBP) have been found to have significant involvement in flower induction, regulation of flowering time, and seed germination in plants (Zhang et al., 2020; Jin et al., 2021). PEBP has been categorized into three subfamilies, namely FLOWERING LOCUS T-like (FT-like), TERMINAL FLOWER 1 (TFL1-like) and MOTHER OF FT and TFL1 (MFT-like). Within the FT-Like subfamily, there are two members, namely Flowering Locus T (FT) and TWIN SISTER OF FT (TSF). FT serves as an integral factor in the flowering pathway and plays a crucial role in the regulation of the flowering process (Adeyemo et al., 2019). Under conditions of extended daylight, the expression of FT is induced, resulting in the formation of the FT-FD complex with FD. This complex then activates the expression of *APETALA1* (*AP1*) and *LEAFY* (*LFY*) genes, thereby facilitating the transition to the flowering stage (Abe et al., 2005; Wigge et al., 2005). In rice, *Hd3a* and *RFT1* play a role in activating the expression of *AP1* homologs, namely *OsMADS14* and *OsMADS15*, thereby promoting flowering (Komiya et al., 2008). Furthermore, *FT* homologs have been extensively identified and studied in various other plant species, such as *Lilium* (Yan et al., 2021), *Cucumis melo L* (Zhang et al., 2020; Zhang and Zhang. 2020), sugarcane (Venail et al., 2022), *Perilla frutescens* (Xu et al., 2022) and *Dendrobium huoshanense* (Song et al., 2021). There is an increasing body of evidence indicating the significant involvement of *FT* genes in the flowering mechanism of plants. In the case of grapes, the expression of *VvFT* serves as an indicator that the developmental transition from juvenile to adult stage has occurred (Carmona et al., 2007). In apples, *MdFT1* and *MdFT2* exhibit distinct expression patterns, yet both possess the capability to initiate early flowering in apple plants (Kotoda et al., 2010). In cassava, the *Arabidopsis FT* gene accelerates flower bud formation and increases lateral branching, and its overexpression also significantly increases flower yield and prolongs flower development life (Adeyemo et al., 2017). Furthermore, the translocation expression of *CsFTL3* in chrysanthemum has been found to stimulate early flowering in both *Arabidopsis* and *chrysanthemum* plants (Oda et al., 2012). These findings suggest that the role of the *FT* gene in flower induction is conserved across various plant species.

MFT, acknowledged as the most ancient constituent of the PEBP family, governs the process of seed germination by means of the abscisic acid (ABA) and gibberellin (GA) signaling pathways in *Arabidopsis* (Xi et al., 2010). The TFL1-like subfamily encompasses TERMINAL FLOWER 1 (TFL1), BROTHER OF FT AND TFL1 (BFT), and CENTRORADIALIS (CEN). Both the TFL1-like and FT-like subfamilies participate in the management of flowering. Nevertheless, in comparison to FT, TFL1 exhibits inhibitory characteristics towards the flowering process (Zhu et al., 2020; Song et al., 2021). In cucumber, the antagonistic interaction between *CsTFL1* and *CsFT*, along with the competitive binding with the CsNOT2a-CsFDP complex, hinders cucumber growth and the development of terminal flowers (Wen et al., 2019). Similarly, in rice, the TFL1-like protein RCN inhibits flower development by competitively binding 14-3-3 and FD proteins to Hd3a/RFT1 (Kaneko-Suzuki et al., 2018). Additionally, the overexpression of *MiTFL1-1* and *MiTFL1-2* genes in mango has been found to significantly impede the flowering in *Arabidopsis* (Gafni et al., 2022).

The pineapple (*Ananas comosus* (L.) Merr.) is a tropical fruit that thrives in tropical and subtropical regions. The timing of its flowering significantly impacts both its yield and the optimal time for harvesting. While the PEBP family of genes is recognized for its crucial role in regulating flowering time and flower development (Jin et al., 2021; Kim et al., 2022), the specific characteristics of the PEBP gene family in pineapple and the specific members involved in the flowering process remain uncertain. This study comprehensively analyzed and identified all members of the PEBP gene family in the pineapple genome, encompassing their phylogenetic relationships, chromosome localization, gene structure, conserved motifs and promoter cis-acting elements. Additionally, considering the pivotal role of the *PEBP* gene in flower development, the expression pattern of AcPEBP and the expression characteristics of FT-like subfamily members were further investigated during flowering. Ultimately, this study provides a theoretical basis for future investigations into the regulatory role of *PEBP* genes in pineapple flowering.

## Materials and methods

### Identification of *AcPEBP* genes in pineapple

Download pineapple gene sequence data from the pineapple genome database (http://pineapple.angiosperms.org/) (Xu et al., 2018). Using the Arabidopsis PEBP protein sequence as a query, the amino acid sequence of Arabidopsis *PEBP* is obtained from the TAIR database (http://www.arabidopsis.org/) (Garcia-Hernandez et al., 2002). The pineapple database was searched with Blastp, and candidate pineapple AcPEBP family gene sequences were identified. After removing redundant sequences, the candidate AcPEBP family genes were submitted to the NCBI database (https://www.ncbi.nlm.nih.gov/cdd) to confirm the presence of the *PEBP* gene domains. The ExPASy website (http://web.expasy.org/protparam/) is to estimate the molecular weight (MW), isoelectric point (PI) and grand average of hydropathicity (GRAVY) for proteins found in pineapples (Wilkins et al., 1999). The subcellular localization prediction was performed using ProtComp v.9.0 (http://linux1.softberry.com/cgi-bin/programs/proloc/protcomppl.pl) (Li et al., 2020).

### Phylogenetic analyses

A multiple sequence alignment was conducted using ClustalX software for pineapple, Arabidopsis, and rice amino acid sequences. Based on 1000 bootstrap analyses performed using MEGA7.0 software with the neighbor-joining (NJ) algorithm (Kumar et al., 2016), the phylogenetic tree was constructed. The tree was visually enhanced and beautified using the chiplot website (https://www.chiplot.online/).

### Conserved motifs and exon-intron structure analysis

Based on the whole-genome GFF annotation file, the analysis of exons and introns was performed using the Gene Structure Display Server 2.0 (http://gsds.cbi.pku.edu.cn). Based on the conserved domain information obtained from NCBI (https://www.ncbi.nlm.nih.gov/), the conserved motifs of the AcPEBP gene family were determined using the MEME online tool (http://meme-suite.org/tools/meme) (Bailey et al., 2009). The results of gene structure and conserved motif analysis were visualized using the TBtools software (Chen et al., 2020).

### *Cis*-element identification and protein interaction prediction analysis

The promoter sequence of the *AcPEBP* gene, spanning 2000bp upstream of the transcription start site, was submitted to the PlantCARE database (http://bioinformatics.psb.ugent.be/webtools/plantcare/html/) for the speculation of cis-regulatory elements in the promoter region (Lescot et al., 2002). The protein-protein interaction (PPI) of AcPEBP was prophesied using the STRING website (https://cn.string-db.org/) (Szklarczyk et al., 2021).

### Pineapple AcPEBP synteny analysis by chromosome location and duplication event

By utilizing the genome sequence and general feature format (GFF) file, the TBtools software was employed to retrieve the chromosomal positions of the pineapple PEBP family genes, enabling the creation of a gene localization information map. Obtain the genome sequences of maize, banana, rice, tomato, grape and *Arabidopsis* by downloading them from the Ensembl plants database (http://plants.ensembl.org/index.html). Collinearity files between each pair of species were obtained using the MCScanX software (http://chibba.pgml.uga.edu/mcscan2/) (Wang et al., 2012). The TBtools software was used to generate the collinearity analysis plot.

### Expression profiling of *AcPEBP* genes by RNA-Seq

The expression data for the *AcPEBP* genes in various pineapple tissues were obtained from the pineapple transcriptome dataset. Furthermore, the transcriptome data for pineapple were publicly deposited in the National Genomics Data Center database (https://ngdc.cncb.ac.cn/gsa/ (accessed on 6 May 2022)), The assigned accession of the submission is CRA006826.The quantification of transcript abundance for each *AcPEBP* gene was performed using the Fragments Per Kilobase of exon per Million mapped reads (FPKM) metric. The visualization of RNA-seq data was achieved by generating heatmaps using TBtools.

### Plant materials and treatments

Pineapple (*Ananas comosus* cv. Comte de Paris) was sourced from the Zhanjiang Subtropical Crop Research Institute in China. Plant materials approximately 30 cm long were selected when the pineapples were around 15 months old. The plants were induced with 30 mL of 200 mg/L^-1^ ethephon, while an equivalent amount of water was used as a control for the untreated materials. Apical buds of pineapple were collected at different time points, including 0 h, 12 h, 1 d, 2 d, 4 d, 1 w, 2 w, 3 w, 4 w, 5 w, 6 w and 7 w after treatment. Each sample was collected with three separate biological replicates. The specimens were rapidly placed in liquid nitrogen and then kept at -80°C in a freezer for later RNA extraction.

### RNA extraction and real-time quantitative PCR analysis

Total RNA was extracted from pineapple samples using a polysaccharide polyphenol plant RNA extraction kit (China Huatuanyang Biotechnology Co., LTD.), and the quality and concentration of total RNA were determined by NanoDropTM One/OneC (Thermo Fisher Scientific, USA), an ultra-fine spectrophotometer. Total RNA First Strand cDNA was synthesized by RevertAid First Strand cDNA Synthesis Kit (Thermo Fisher Scientific, USA). qRT-PCR was performed using the SYBR Green qPCR Master MIX Kit (Thermo Fisher Scientific, USA). Employing the *AcActin* gene as the internal reference gene, qRT-PCR was performed to calculate the relative expression level of the *AcPEBP* gene through the 2^-ΔΔCt^ method (Rao et al., 2013). Each sample was replicated three times.

### Subcellular localization of PEBP proteins

To validate the subcellular localization of the AcPEBP protein in plant cells, the full-length sequence of *AcFT4* was cloned into the pCAMBIA2300-GFP vector to generate the fusion construct (2300 GFP-*AcFT4*). The fusion construct and control vector were transformed into the Agrobacterium tumefaciens strain GV3101 (ANGYUBio, China). Subsequently, they were used for transformation into healthy tobacco leaves. After 2-3 d, the transient expression of *AcFT4*-GFP was observed using a confocal laser scanning microscope (Zeiss, Germany).

### Transcriptional activity of PEBP proteins

The pGBKT7-*AcFT4* fusion vector was constructed and transferred into the yeast strain AH109 for the analysis of AcFT4 protein’s transcriptional activation activity (AngYuBio, China). The transformed colonies were streaked on selective medium SD/−Trp and incubated at 30°C for 48-72 h. Subsequently, the colonies were further selected on SD/−Trp/−His/−Ade medium, and X-α-Gal was added to observe if the AcFT4 protein exhibited transcriptional activation activity.

### Expression vector construction and plant transformation

The agrobacterium containing the recombinant plasmid was introduced into *Arabidopsis* flowers through the floral dip method (Zhang et al., 2006). *Arabidopsis* plants were cultured at room temperature (22°C) until they matured. Subsequently, transgenic positive plants were selected on MS medium supplemented with 50 mg/mL kanamycin. Healthy and green *Arabidopsis* plants were chosen for rapid PCR-based DNA screening to confirm positive transgenic individuals. The selected positive seedlings were further screened for T3 generation, to obtain homozygous transgenic lines, and the phenotypes of the transgenic homozygous lines were observed.

## Results

### Characterization and identification of PEBP proteins in pineapple

The BLASTP search was conducted in the pineapple genome database, utilizing the Arabidopsis PEBP protein as the query. Following the elimination of redundant members, a total of 11 *AcPEBP* genes were identified and subsequently named based on their resemblance to homologous genes in Arabidopsis. These genes encompass *AcFT1*-*AcFT7*, *AcTFL1a*-*AcTFL1b* and *AcMFT1*-*AcMFT2*. As depicted in **Table 1**, the AcPEBP family consists of 11 members, exhibiting varying protein lengths that range from 110 amino acids (AcMFT2) to 238 amino acids (AcFT6). The molecular weights of the proteins in this family vary from 12.34 kDa (AcMFT2) to 26.93 kDa (AcFT6). The isoelectric points of these proteins range from 6.96 (AcMFT1) to 9.61 (AcFT1). All members of this protein family exhibit a negative GRAVY value, indicating their hydrophobic nature. Subcellular localization prediction analysis revealed that, with the exception of AcMFT1, which is localized in Chloroplasts and Cytoplasm, the other AcPEBP member proteins are predicted to be located in the nucleus. Additionally, AcFT1 and AcMFT2 are also distributed in the Cytoplasm.

**Table 1:** PEBP gene family physicochemical characterization in pineapple.

Gene	GeneBank ID	Protein/AA	MW (kDa)	pI	GRAVY	Subcellular localization
*AcFT1*	Aco004292.1.v3	176	19.80	9.61	-0.261	Cytoplasm. Nucleus.
*AcFT2*	Aco004692.1.v3	222	25.26	9.03	-0.627	Nucleus.
*AcFT3*	Aco010683.1.v3	215	24.27	7.83	-0.101	Nucleus.
*AcFT4*	Aco010684.1.v3	177	19.84	6.96	-0.214	Nucleus.
*AcFT5*	Aco006026.1.v3	180	20.18	8.74	-0.280	Nucleus.
*AcFT6*	Aco003470.1.v3	238	26.93	8.70	-0.206	Nucleus.
*AcFT7*	Aco008070.1.v3	231	25.92	8.91	-0.223	Nucleus.
*AcTFL1a*	Aco031443.1.v3	173	19.37	9.00	-0.360	Nucleus.
*AcTFL1b*	Aco016718.1.v3	147	16.75	9.09	-0.463	Nucleus.
*AcMFT1*	Aco006778.1.v3	184	20.81	6.96	-0.296	Chloroplasts. Cytoplasm.
*AcMFT2*	Aco012712.1.v3	110	12.34	9.30	-0.462	Cytoplasm. Nucleus.

### Phylogenetic analysis

**Figure 1:**
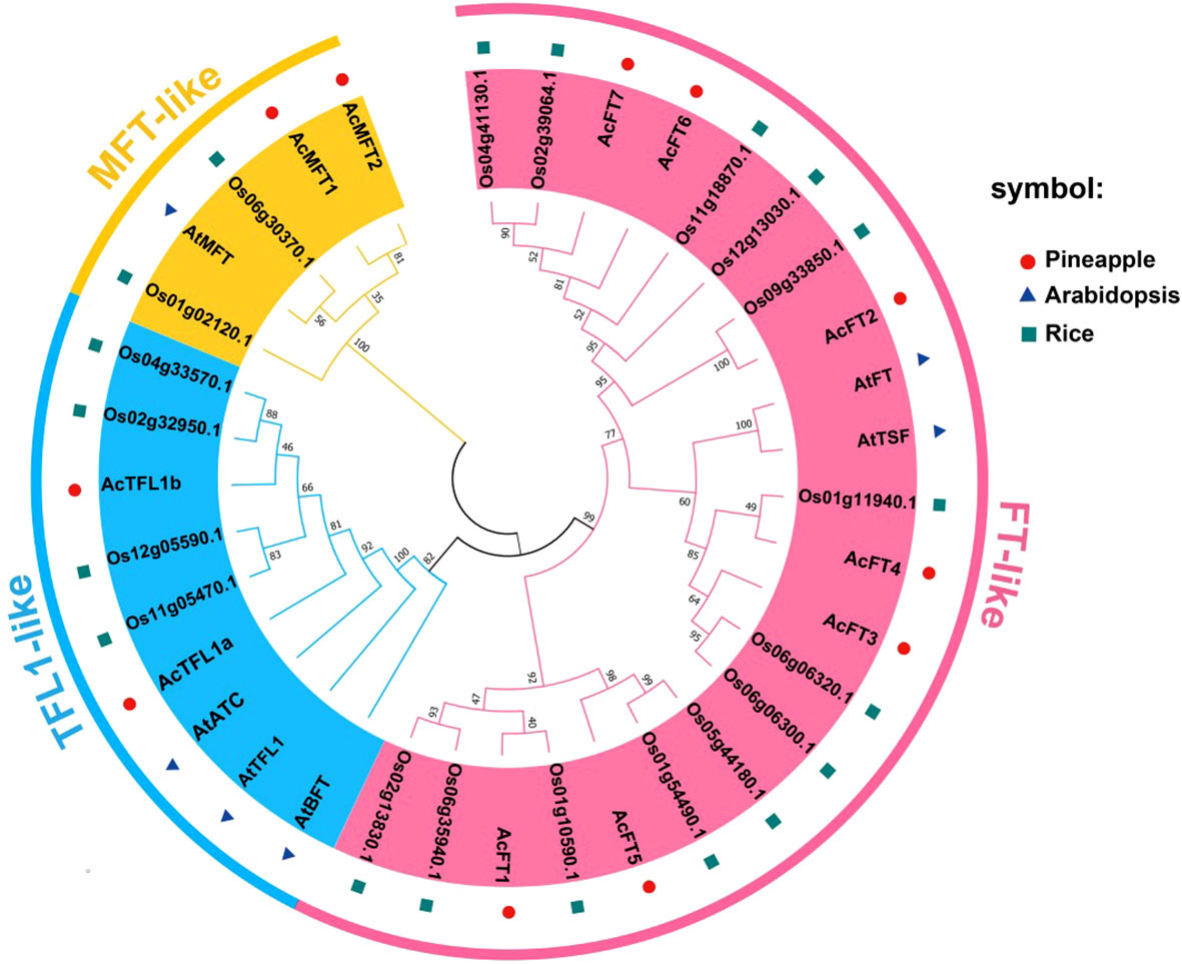
The phylogenetic analysis of PEBP proteins among pineapple, *Arabidopsis* and rice. The phylo-genetic tree was constructed using MEGA7.0 program. (The neighbor-joining (NJ) algorithm, bootstrap value = 1000). The phylogenetic connections and structural characteristics of the 11 AcPEBP genes.

In order to examin the evolutionary relationships among AcPEBP members, a phylogenetic tree (**Figure 1**) was generated using the neighbor-joining technique with MEGA software. This analysis was based on the full-length amino acid sequences obtained from pineapple, Arabidopsis, and rice. The resulting phylogenetic tree classified the 11 AcPEBP proteins, 5 AtPEBP proteins, and 19 OsPEBP proteins into three distinct subfamilies, namely FT-like, TFL-like and MFT-like, as depicted in **Figure 1**. Among the AcPEBP family members, the FT-like subfamily displayed the greatest abundance of members, with a total of 7 AcPEBP proteins. In contrast, both the TFL-like and MFT-like subfamilies consisted of only 2 members each.

### Gene structures, conserved domains, and motif analysis

**Figure 2:**
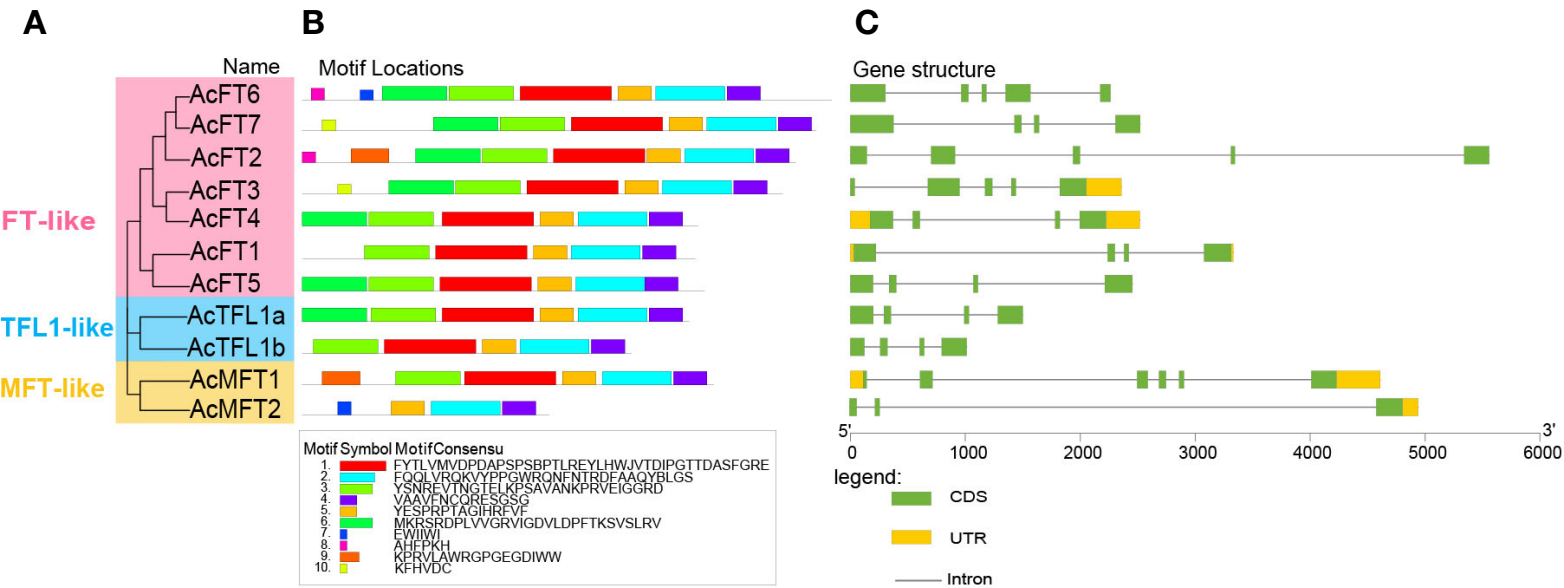
Phylogenetic connections and structural characteristics of the 11 *AcPEBP* genes. **(A)** Phylogenetic analysis using the Neighbor-Joining method for the PEBP family in pineapple. **(B)** The patterns of predicted motifs within the AcPEBP proteins. **(C)** Gene structures of PEBP family genes in pineapple. Yellow icons represent untranslated regions (UTR), green icons represent coding sequences (CDS), and lines connecting UTR and CDS represent introns (a noncoding segments of a gene or mRNA).

To acquire a more thorough comprehension of the AcPEBP gene members, we utilized the MEME website to forecast the conserved motifs present within the AcPEBP family. As illustrated in **Figure 2**, the number of conserved motifs in each AcPEBP protein ranges from Motif 4 to Motif 8. All members include motifs Motif 2, Motif 4, and Motif 5, while a majority of the members exhibit motifs 1 through 5 (**Figure 2B**). This implies that the AcPEBP protein demonstrates a notable degree of conservation among its constituents. **Figure 2C** shows that the quantity of introns in the AcPEBP gene family, ranging from 2 to 5. Among them, the MFT subfamily exhibits the highest number of introns in *AcMFT1*, reaching 5, while *AcMFT2* within the same subfamily displays the lowest count, with only 2 introns. Furthermore, other subfamily members share similarities in terms of the amount of introns and exons, suggesting a potential correlation between genetic structure and specific biological functions, regulatory pathways, or evolutionary relationships (Liu et al., 2021).

### *Cis*-element analysis of AcPEBPs

**Figure 3:**
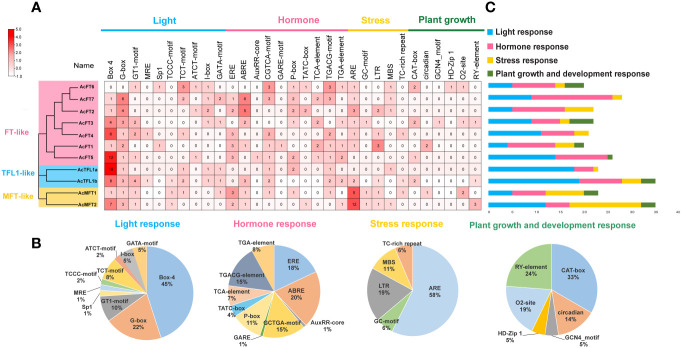
Analysis of cis-elements within the promoters of *AcPEBP* genes. **(A)** The *cis*-element analysis of *PEBP* promoter region. **(B)** The count of various types of elements in *AcPEBP* promoters was tallied and is represented using different colors. **(C)** The pie chart shows the proportion of each *c*is-element of the four types of response elements.

For the purpose of comprehending the potential roles and expression regulatory mechanisms of the *AcPEBP* gene, cis-acting elements located within the gene’s promoter region (upstream 2 kb region) were predicted using the PlantCARE website (**Figure 3**). A total of 27 cis-acting elements were identified, encompassing 7 light-responsive elements, 9 plant hormone-responsive elements, 5 stress-related elements and 6 growth and development-responsive elements. The plant hormone-responsive elements, including encompassing gibberellin-responsive elements (TATC-box, P-box and GARE motif), auxin-responsive elements (TGA-element and AuxRR core), salicylic acid-responsive elements (TCA-element), abscisic acid-responsive elements (ABRE), as well as CGTCA and TGACG motifs involved in MeJA (methyl jasmonate) responses, are the most abundant. Additionally, the promoters of 10 *AcPEBP* gene family members contain light-responsive elements Box 4 and G-box. This observation suggests that the AcPEBP gene family may exhibit responsiveness to both hormonal and light signals.

### Chromosomal position and gene duplication of the *AcPEBP* gene

**Figure 4:**
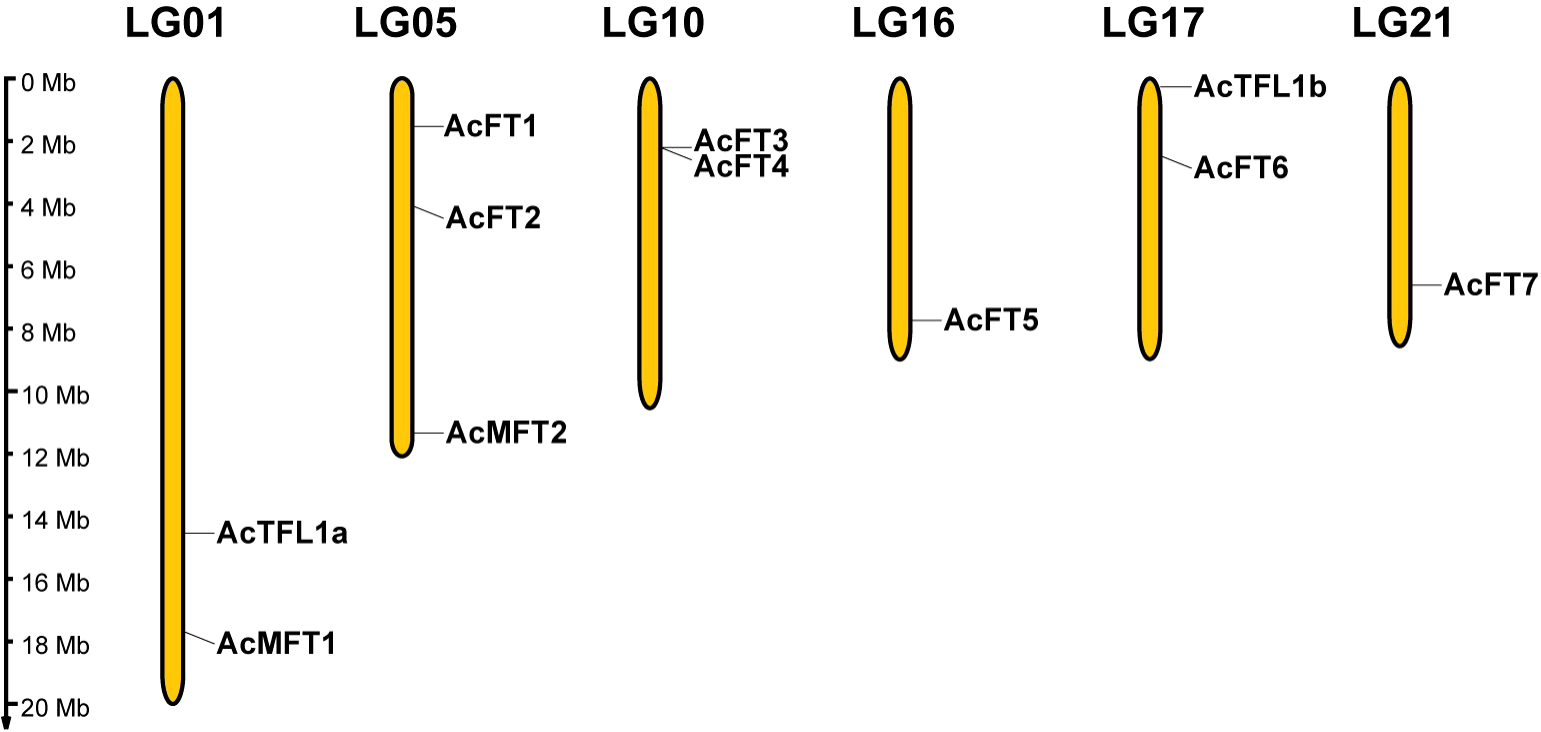
Distribution of AcPEBP genes on the pineapple chromosomes. The yellow vertical lines indicate the pineapple chromosome (Chr), the number is above the Chr. The left scale bar is the length of the chromosome.

In order to comprehend the distribution characteristics and gene replication of the PEBP gene, the genomic annotation information of pineapple was employed to ascertain the chromosomal localization of the *AcPEBP* gene. As depicted in **Figure 4**, the 11 genes exhibit an uneven distribution across 6 chromosomes, constituting approximately 1/4 of the 25 chromosomes present in pineapple. Notably, chromosome 5 harbors the highest number of three *AcPEBP* genes; whereas chromosomes 1, 10 and 17 each contain two *AcPEBP* genes, there is only one *AcPEBP* gene on chromosome 16.

**Figure 5:**
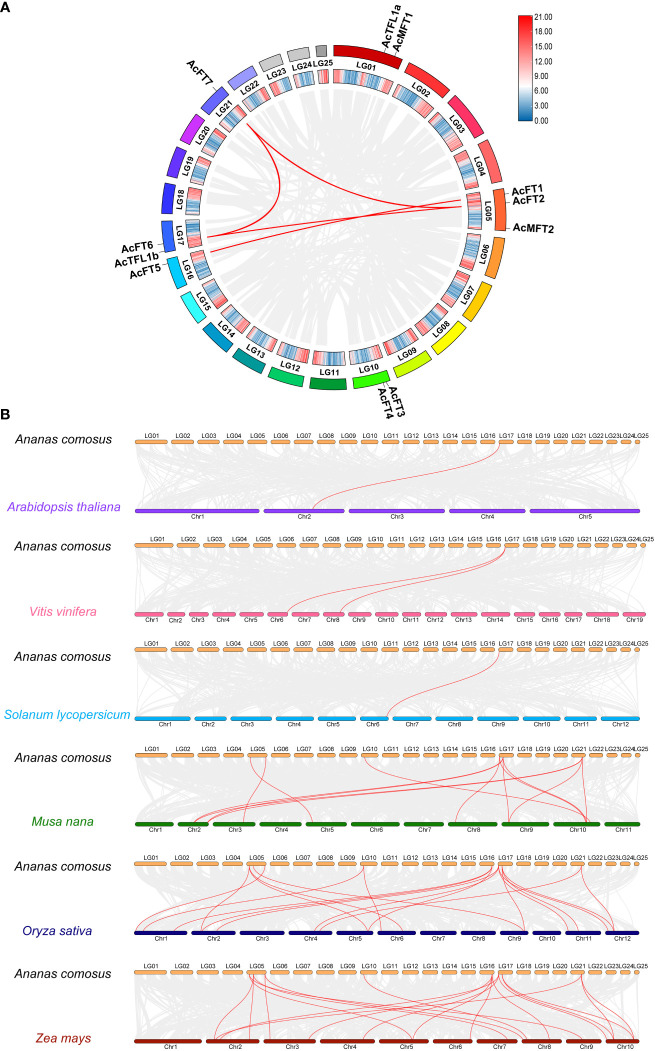
Duplication analysis and synlinearity analysis. **(A)** Duplicate analysis of *AcPEBP* gene in pineapple. **(B)** Synteny analysis of *AcPEBP* gene between pineapple and other plants (*Arabidopsis*, grape, tomato, banana, rice and corn). The red line indicates homologous gene pairs.

To unravel the expansion mechanism of the pineapple PEBP gene family, gene collinearity analysis was performed. The results, as shown in **Figure 5**, revealed that 11 *AcPEBP* genes were involved in 4 segmental duplication events (*AcFT1*/*AcFT5*, *AcFT6*/*AcFT7*, *AcFT2*/*AcFT7*, *AcFT2*/*AcTFL2*), as well as 1 tandem duplication event (AcFT3/AcFT4) (**Figure 5A**). With the exception of *AcFT2*/*AcTFL2*, the majority of genes implicated in the duplication events belong to the FT-like subfamily. This observation suggests that gene duplication appears to be the primary mechanism driving the expansion of the pineapple FT-like subfamily. To enhance comprehension of the evolutionary trajectory and source of the AcPEBP gene family, collinearity maps were generated comparing pineapple with three dicotyledonous plants (*Arabidopsis*, grape and tomato), as well as three monocotyledonous plants (banana, maize and rice) (**Figure 5B**). Pineapple displays 13, 20 and 19 homologous gene pairs with banana, maize and rice, respectively, notably higher count compared to the homologous gene pairs observed between pineapple and *Arabidopsis* (1), tomato (1) and grape (2). This phenomenon can be attributed to the fact that pineapples pertain to the category of monocotyledonous plants, thereby establishing a more intimate evolutionary connection with other species falling under the same category, while exhibiting a comparatively distant evolutionary association with dicotyledonous species.

### AcPEBP protein interaction network

**Figure 6:**
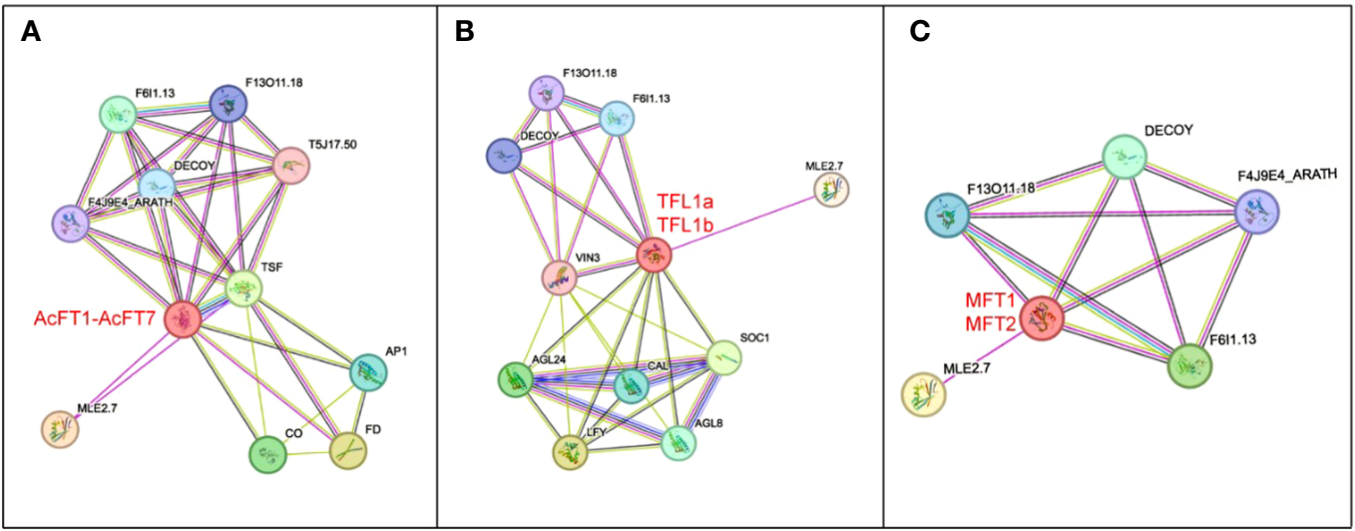
Depicts the predicted protein-protein interaction regulatory network of AcPEBP. **(A-C)** Respectively represent the network predictions for AcFTs, AcTFLs and AcMFTs, along with their interacting proteins.

The construction of the protein-protein interaction network of AcPEBP was based on the interactions of homologous proteins in *Arabidopsis thaliana* (**Figure 6**). Noteworthy findings were observed within this network. Specifically, in the FT interaction network (**Figure 6A**), CO, FD, and AP1 as crucial components in the *Arabidopsis* FT floral-dependent regulation (Komeda, 2004). Additionally, AcTFL1 may interact with VIN3, CAL, AGL8, AGL24, SOC1, and LFY (**Figure 6B**). The regulation of these genes likely entails specific nodes within the flowering regulatory network that synchronize the timing of flowering and orchestrate the progression of the flowering process (Azpeitia et al., 2021). DECOY and MLE2.7 appear in the interaction networks of three subfamilies (**Figure 6**), suggesting their potential significance in plant growth, development, and other biological processes. Consequently, conducting a thorough investigation into the pivotal factors governing the regulation of FT will yield a more comprehensive comprehension of the flowering regulatory mechanism exhibited by PEBP family constituents in pineapple.

### Expression profiles of *AcPEBP* in different tissues in pineapple

**Figure 7:**
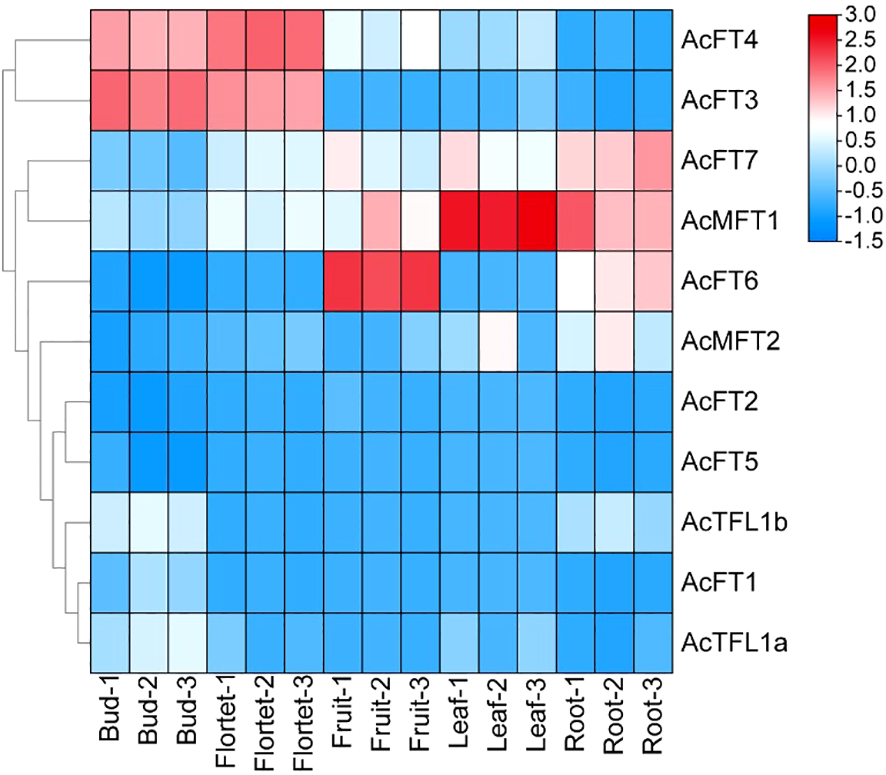
Expression profile of *AcPEBP* genes in different tissues of pineapple. The labels of the X-axis represent the different organs of the pineapple. The Y-axis refers to the deduced FPKM value normalized with Log2.

To explore the expression patterns of the AcPEBP gene family across various tissues, transcriptomic data to determine the expression levels of *PEBP* genes in pineapple buds, flowers, fruits, leaves and roots. Our findings revealed distinct tissue specificity in the expression of AcPEBP family members (**Figure 7**). Notably, *AcFT3* and *AcFT4* exhibited significantly higher expression levels in flowers and buds compared to other tissue sites. Additionally, *AcFT6* and *AcMFT1* displayed the highest expression levels in fruits and leaves, respectively;*AcMFT1* and *AcFT7* also exhibited elevated expression levels in roots. In addition to these members, *AcFT1*, *AcFT2*, *AcFT5*, *AcTFL1a*, *AcTFL1b* and *AcMFT2* are almost not expressed. Consequently, it can be inferred that *AcFT3* and *AcFT4* likely assume a significant function in the process of flowering and flower development in pineapple.

### Expression patterns of *AcPEBP* during flower induction and the flowering process in pineapple

**Figure 8:**
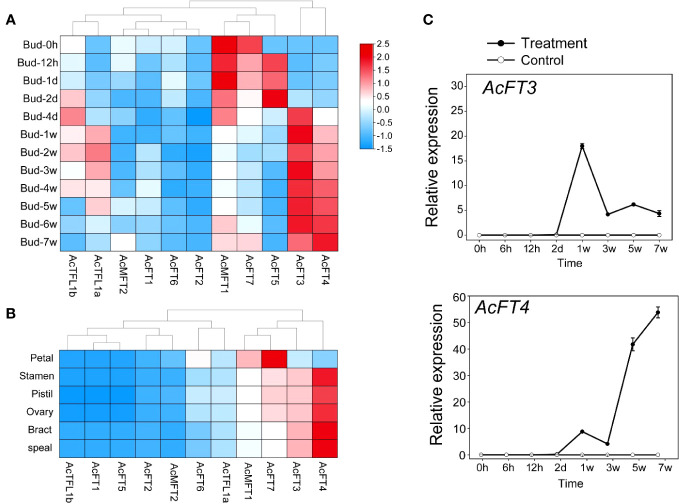
Expression profile of AcPEBP genes in flower induction and flowering process. **(A)** A heatmap illustrating the transcriptional levels of AcPEBP genes as determined by RNA-seq. **(B)** Expression pattern analysis of AcPEBP genes across various floral organs in pineapple. **(C)** Validation of AcPEBP gene expressions using qRT-PCR.

To enhance the credibility of *AcPEBP* in flower induction and flowering processes, a thorough investigation was conducted to assess the expression patterns of *AcPEBP* at different stages of flower development. As illustrated in **Figure 8**, the *AcPEBP* gene exhibited distinct patterns of expression. However, of greater interests to us is the observation that the members exhibiting elevated expression levels during the late stages of flower development in pineapple are predominantly concentrated within the FT-like subfamily. For example, during the course of the flowering process, the expression levels of *AcFT3* and *AcFT4* exhibited a gradual increment in conjunction with the development of flower buds (**Figure 8A**). qRT-PCR findings indicated that the expression levels of *AcFT3* and *AcFT4* genes in flower bud differentiation gradually increased after 4 days of ethethylene treatment and culminating in a peak at 5 to 7 weeks (**Figure 8C**). Furthermore, it is noteworthy that the fifth week following ethylene catalysis emerges as a pivotal phase for flower organ differentiation. Subsequently, an in-depth examination of the expression patterns of the *AcPEPB* gene in various flower organs of the pineapple was conducted (**Figure 8B**). The expression levels of *AcFT7*, *AcFT3* and *AcFT4* in different flower organs of pineapple were significantly increased.The concordance between the RNA-seq data and the qRT-PCR analysis results provides additional validation for the experimental findings. These findings suggest that the FT-like subfamily within the PEBP family may have a significant role in the pineapple flowering process.

### Localization within cells and transcriptional activity of AcFT4

**Figure 9:**
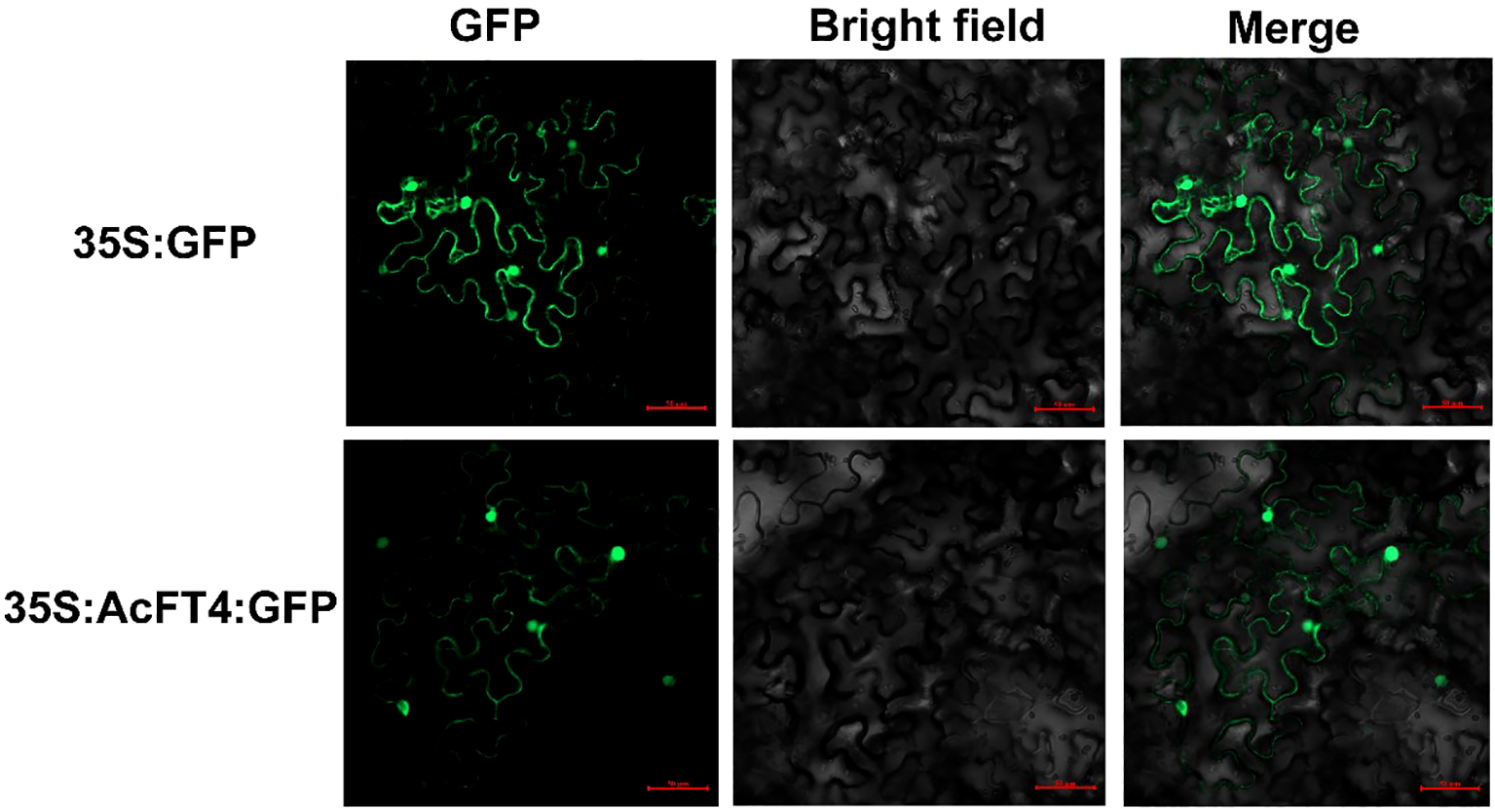
Subcellular localization of *AcPEBP* genes. Each candidate gene was individually inserted into the pCAMBIA2300-GFP vector. The empty vector was used as the control.

**Figure 10:**
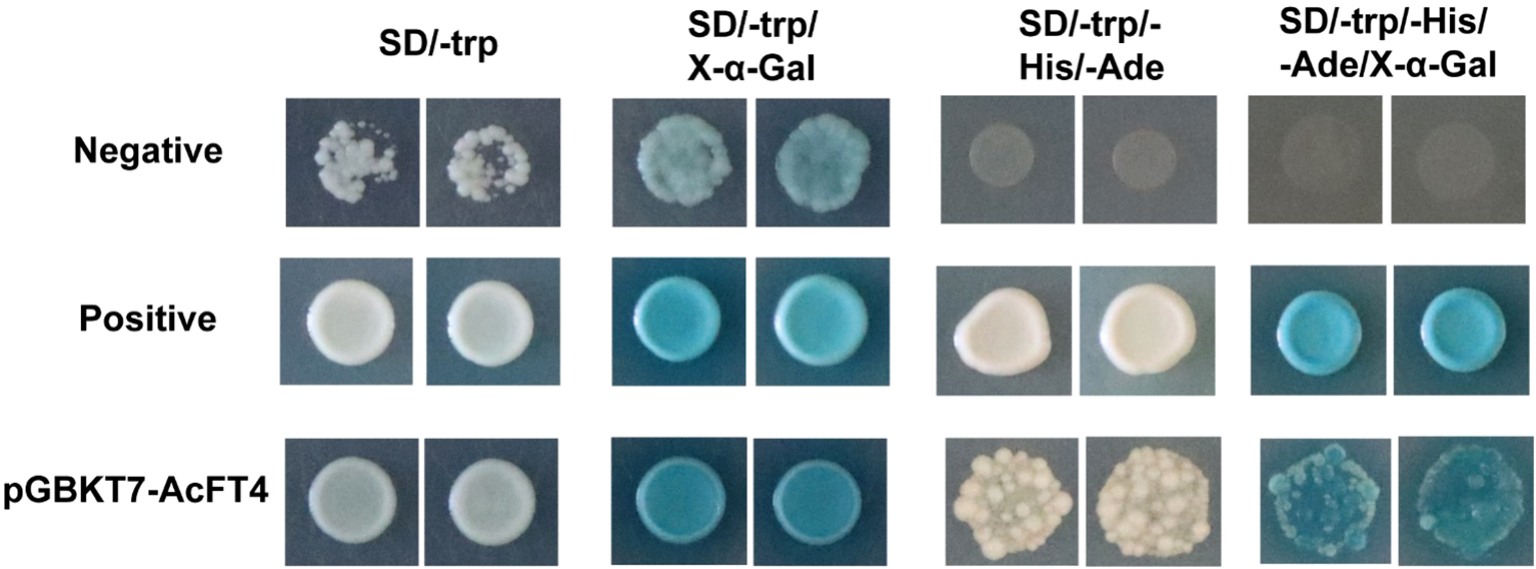
Assessment of the transcriptional activity of AcFT4 in yeast. The negative control involved the transformation of the empty vector pGBKT7 into yeast. The positive control consisted of the co-transformation of pGADT7-T and pGBKT7-53 into yeast.

To elucidate the potential functions of *AcFT4* in the transcriptional regulatory system, a recombinant *AcFT4*-GFP protein was synthesized and temporarily expressed in the leaves of tobacco plant (*Nicotiana benthamiana*). GFP originating from the *AcFT4*-GFP fusion protein were observed in both the nucleus and peripheral cytoplasm (**Figure 9**), which is consistent with the results observed in other species (Harig et al., 2012; Liu et al., 2022). To investigate whether the AcFT4 protein has transcriptional activity, the coding sequence of AcFT4 was integrated with the GAL4 DNA-binding domain encoding sequence in the pGBKT7 vector. The resulting recombinant construct was introduced into the yeast strain AH109. The findings revealed robust growth of yeast colonies on the SD/-Trp medium (**Figure 10**). In addition to negative controls, blue yeast colonies transformed with pGBT7-*AcFT4* were observed on SD/-Trp/-His/-Ade medium. This observation indicates that AcFT4 may indeed participate in the transcriptional regulatory system.

### Over-expression of *AcFT4* in *Arabidopsis* strongly accelerated flowering

**Figure 11:**
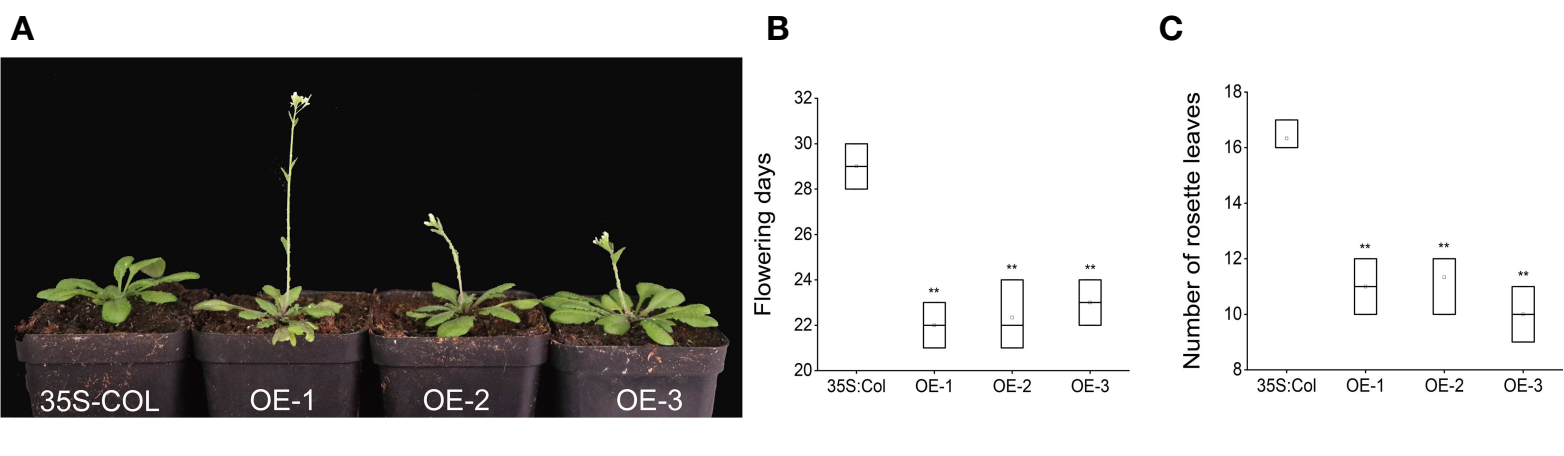
*AcPEBP* plants display an early flowering phenotype. **(A)** Transgenic lines. **(B)** Flowering days. **(C)** Number of rosette leaves. Values represent the mean ± SD of three independent biological replicates. The asterisk denotes a significantly different compared with controls (**P < 0.05, based on t-test).

The 35S::*AcFT4* construct was introduced into wild-type *Arabidopsis thaliana* to assess whether *AcFT4* induces flower development. The empty vector with the CaMV35S promoter was transformed into *Arabidopsis* plants as the negative control (35S-COL). After kanamycin screening and PCR identification, three T3 transgenic plants were selected for further analysis, taking into consideration the expression level of *AcFT4*. Compared with the controls, all transgenic strains showed different degrees of early flowering (**Figures 11A–C**). Transgenic plants overexpressing *AcFT4* had an earlier flowering time of 6-7 days and had 5-6 fewer rosette leaves at flowering (**Figures 11B, C**). These data suggest that overexpression of *AcFT4* can promote flowering in transgenic *Arabidopsis thaliana*.

## Discussion

The PEBP family in plants ethanolamine-binding proteins that are highly conserved. These genes are crucial in regulating various processes such as the transition to flowering, seed development and dormancy, and the formation of inflorescence structure in higher plants (Yue et al., 2021; Chen F. et al., 2021; Périlleux et al., 2019). Despite the extensive research on the PEBP family in other plant species, the function of this family in pineapples remains inadequately investigated. Therefore, there is a pressing need for further investigation into the function of the AcPEBP family in pineapples. In this study, 11 PEBP genes were discovered within the pineapple genome database. Similar to *Arabidopsis* and rice, the PEBP gene family within the pineapple genome was also divided into three subfamilies: FT-like, TFL1-like and FT-like. This is also consistent with the classification of fruit tree crops such as grapes (Carmona et al., 2007). However, there are significant differences in the number of PEBP family members between species, potentially attributed to gene duplication or deletion events during the course of evolution. A previous study suggested that exon and intron structures were involved in the evolution of plant species (Zhang and Li, 2017). In particular, most genes in the FT/TFL1 subfamily predominantly features a gene structure comprising four exons and three introns (**Figure 2C**), and they show a similar pattern of conserved motif distribution. This suggests that these genes are conserved in evolution. However, in contrast to the FT/TFL1 subfamily, the *AcMFT2* gene had five introns, while the *AcMFT1* gene had only two introns. The presence of exon-intron acquisition and deletion within the MFT-like subgroup of pineapple, along with substantial alterations in the conserved pattern, suggests potential functional diversity among MFT-like genes. (Xi and Yu, 2010; Song et al., 2020).

At present, it is generally believed that the expression pattern of genes is typically associated with their biological functions (Dong et al., 2020). Previous studies have demonstrated that *Arabidopsis AtFT* gene exhibits expressed in both reproductive and vegetative organs (Kobayashi et al., 1999). *RoFT* gene of rose is expressed in flower bud tips and buds (Remay et al., 2009). The expression of the *VvFT* gene was detected during the development of grape inflorescence (Carmona et al., 2007). In apples, *MdFT1* primarily assumes a significant role in apple fruit development, of whereas *MdFT2* is predominantly expressed in the reproductive organs of apple, including flower buds and young fruits. The *NtFT5* gene in tobacco is expressed in tobacco flowers (Wang et al., 2018). In this study, the expression pattern of the AcPEBP gene family exhibited certain tissue specificity. Specifically, *AcFT7* and *AcFT6* were highly expressed in the root and fruit of pineapple, respectively, and *AcMFT1* exhibited high expression levels in the root and leaf tissues. These genes likely play pivotal roles within the contexts of roots, leaves and fruits. It is worth noting that *AcFT3* and *AcFT4* not only exhibited higher transcription levels in flower buds compared to other tissues, but also displayed elevated expression levels in flowers (**Figure 7**). After that, the expression of *AcFT3* and *AcFT4* was observed to be significantly specific across five distinct flower organs of pineapple (**Figure 8B**). This suggests that the *AcFT* genes potentially exert a significant regulatory on the process of flower bud formation and flowering in pineapple. In order to further verify the results, the expression of the *FT* gene in pineapple flower buds was analyzed assessed through qRT-PCR. The results showed that the expression level of *AcFT4* began to increase within just 4 days after ethylene treatment and reached a peak in late flower development (**Figure 8C**). This indicates that members of the pineapple PEBP family might play an important role in the flowering process of pineapple.

Simultaneously, examination of promoter cis-acting elements has suggested the possible participation of light-responsive and hormone-responsive within the intricate flowering regulatory network. (**Figure 6**). This phenomenon has been previously observed in various plant species. The peanut FT-like gene exhibits higher elevated expression levels in short-day conditions and is involved in the regulation of flowering time under short-day conditions (Jin et al., 2019).In *Dendrobium huoshanense*, gibberellin acid (GA) induces strong expression of *DhFT3* and *DhFT10*, while the expression of *DhTFL1* swiftly declines following GA treatment. This may have different regulatory roles in flowering control (Song et al., 2021). The short-day plant *Pharbitis nil FT* homolog *PnFT2* may be stimulated through the application of salicylic acid (SA) (Wada et al., 2010). In a recent groundbreaking discovery, Chen Y. et al. revealed that *ERF1* functions as a regulatory factor in ethylene signaling, directly suppressing the expression of *FT* in *Arabidopsis* during a complex flowering cascade, delaying flowering (Chen Y. et al., 2021). Interestingly, treatment of pineapple plants with the ethylene-releasing agent ethephon has been demonstrated to induce early flowering of pineapples (Espinosa et al., 2017). Therefore, we posit that the flowering pathway in pineapples may differ from that of the model plant *Arabidopsis*.

FT-like genes in plants play a crucial role in regulating flowering (Takagi et al., 2023). This study demonstrated that the overexpression of *AcFT4* in *Arabidopsis* resulted in early flowering (**Figure 11**). Transgenic lines showed flowering time 6-7 days earlier than empty vector plants with CaMV35S promoters. This finding suggests that *AcFT4* acts as a flower promoting factor, consistent with other species. For instance, the ectopic expression of *ZCN8* in maize accelerates flowering in transgenic *Arabidopsis* (Danilevskaya et al., 2008). Overexpression of *MiFT1*, *MiFT2* and *MiFT3* in mango has been found to induce early flowering phenotypes in *Arabidopsis* (Fan et al., 2020). In strawberries, *FveFT2* is a non-photoperiodic florigen that allows short-day plants to flower, and its excessive expression greatly enhances the flowering phenomenon (Gaston et al., 2021). In addition to the function of FT itself, key components CO, FD and LFY in the known flowering regulation network of *FT* genes were found in the protein interaction prediction network (**Figure 6**) (Blümel et al., 2015). These genes collaborate within signaling pathway, collectively orchestrating the process of flowering. Nevertheless, whether the *FT* gene in pineapple functions through these flowering regulatory factors still requires further investigation. This will contribute to a more comprehensive understanding of the molecular mechanisms of PEBP members in the pineapple flowering signaling pathway.

## Conclusions

In this study, 11 members of the PEBP family were identified from the pineapple genome annotation file. Analysis of *cis*-acting elements suggests that *AcPEBP* genes may be influenced by a complex network of hormonal and light regulation. The analysis of gene expression showed that *AcFT3* and *AcFT4* were specifically highly expressed in the later stages of flower development and in floral organs, suggesting that these genes may be involved in the process of pineapple flowering induction. Following this, the subcellular localization and transcriptional activation activities of AcFT4 were subsequently confirmed. AcFT4 exhibits transcriptional activity and is localized in various cellular compartments, including the nucleus and peripheral cytoplasm. The overexpression of *AcFT4* in transgenic *Arabidopsis thaliana* resulted in an expedited flowering process. In addition, important components of floral-dependent regulation of FT, CO, LFY and AP1, were found in the protein interaction network. In conclusion, the genomic analysis of AcPEBP family provides a new basis for the study of flower development related functions of PEBP family members in pineapple.

## Data availability statement

The original contributions presented in the study are included in the article/supplementary material. Further inquiries can be directed to the corresponding authors.

## Author contributions

HZ: Writing – review & editing, Funding acquisition. XZ: Data curation, Software, Writing – original draft. YO: Conceptualization, Writing – review & editing. LZ: Data curation, Investigation, Resources, Software, Writing – review & editing. ZL: Investigation, Resources, Writing – review & editing. YW: Writing – review & editing.

## References

[B1] Abe M. Kobayashi Y. Yamamoto S. Daimon Y. Yamaguchi A. Ikeda Y.. (2005). FD, a bZIP protein mediating signals from the floral pathway integrator FT at the shoot apex. Science 309, 1052–1056. doi: 10.1126/science.1115983 16099979

[B2] Adeyemo O. S. Chavarriaga P. Tohme J. Fregene M. Davis S. J. Setter T. L. (2017). Overexpression of Arabidopsis FLOWERING LOCUS T (FT) gene improves floral development in cassava (Manihot esculenta, Crantz). PloS One 12, e0181460. doi: 10.1371/journal.pone.0181460 28753668 PMC5533431

[B3] Adeyemo O. S. Hyde P. T. Setter T. L. (2019). Identification of FT family genes that respond to photoperiod, temperature and genotype in relation to flowering in cassava (Manihot esculenta, Crantz). Plant Reprod. 32, 181–191. doi: 10.1007/s00497-018-00354-5 30543044 PMC6500508

[B4] Azpeitia E. Tichtinsky G. Le Masson M. Serrano-Mislata A. Lucas J. Gregis V.. (2021). Cauliflower fractal forms arise from perturbations of floral gene networks. Science 373, 192–197. doi: 10.1126/science.abg5999 34244409

[B5] Bailey T. L. Boden M. Buske F. A. Frith M. Grant C. E. Clementi L.. (2009). MEME SUITE: tools for motif discovery and searching. Nucleic Acids Res. 37, W202–W208. doi: 10.1093/nar/gkp335 19458158 PMC2703892

[B6] Blümel M. Dally N. Jung C. (2015). Flowering time regulation in crops—what did we learn from Arabidopsis? Curr. Opin. Biotechnol. 32, 121–129. doi: 10.1016/j.copbio.2014.11.023 25553537

[B7] Carmona M. J. Calonje M. Martínez-Zapater J. M. (2007). The FT/TFL1 gene family in grapevine. Plant Mol. Biol. 63, 637–650. doi: 10.1007/s11103-006-9113-z 17160562

[B8] Chen C. Chen H. Zhang Y. Thomas H. R. Frank M. H. He Y.. (2020). TBtools: an integrative toolkit developed for interactive analyses of big biological data. Mol. Plant 13, 1194–1202. doi: 10.1016/j.molp.2020.06.009 32585190

[B9] Chen F. Li Y. Li X. Li W. Xu J. Cao H.. (2021). Ectopic expression of the Arabidopsis florigen gene FLOWERING LOCUS T in seeds enhances seed dormancy via the GA and DOG1 pathways. Plant J. 107, 909–924. doi: 10.1111/tpj.15354 34037275

[B10] Chen Y. Zhang L. Zhang H. Chen L. Yu D. (2021). ERF1 delays flowering through direct inhibition of FLOWERING LOCUS T expression in Arabidopsis. J. Integr. Plant Biol. 63, 1712–1723. doi: 10.1111/jipb.13144 34152677

[B11] Danilevskaya O. N. Meng X. Hou Z. Ananiev E. V. Simmons C. R. (2008). A genomic and expression compendium of the expanded PEBP gene family from maize. Plant Physiol. 146, 250–264. doi: 10.1104/pp.107.109538 17993543 PMC2230559

[B12] Dong L. Lu Y. Liu S. (2020). Genome-wide member identification, phylogeny and expression analysis of PEBP gene family in wheat and its progenitors. PeerJ 8, e10483. doi: 10.7717/peerj.10483 33362967 PMC7747686

[B13] Espinosa M. E. Á. Moreira R. O. Lima A. A. Ságio S. A. Barreto H. G. Luiz S. L. P.. (2017). Early histological, hormonal, and molecular changes during pineapple (Ananas comosus (L.) Merrill) artificial flowering induction. J. Plant Physiol. 209, 11–19. doi: 10.1016/j.jplph.2016.11.009 27988471

[B14] Fan Z. Y. He X. H. Fan Y. Yu H. X. Wang Y. H. Xie X. J.. (2020). Isolation and functional characterization of three MiFTs genes from mango. Plant Physiol. Biochem. 155, 169–176. doi: 10.1016/j.plaphy.2020.07.009 32768921

[B15] Gafni I. Rai A. C. Halon E. Zviran T. Sisai I. Samach. A.. (2022). Expression profiling of four mango FT/TFL1-encoding genes under different fruit load conditions, and their involvement in flowering regulation. Plants (Basel) 11, 2409. doi: 10.3390/plants11182409 36145810 PMC9506463

[B16] Garcia-Hernandez M. Berardini T. Z. Chen G. Crist D. Doyle A. Huala E.. (2002). TAIR: a resource for integrated Arabidopsis data. Funct. Integr. Genomics 2, 239–253. doi: 10.1007/s10142-002-0077-z 12444417

[B17] Gaston A. Potier A. Alonso M. Sabbadini S. Delmas F. Tenreira T.. (2021). The FveFT2 florigen/FveTFL1 antiflorigen balance is critical for the control of seasonal flowering in strawberry while FveFT3 modulates axillary meristem fate and yield. New Phytologist 232, 372–387. doi: 10.1111/nph.17557 34131919 PMC8519138

[B18] Harig L. Beinecke F. A. Oltmanns J. Muth J. Müller O. Rüping B.. (2012). Proteins from the FLOWERING LOCUS T-like subclade of the PEBP family act antagonistically to regulate floral initiation in tobacco. Plant J. 72, 908–921. doi: 10.1111/j.1365-313X.2012.05125.x 22889438

[B19] Jin S. Nasim Z. Susila H. Ahn J. H. (2021). Evolution and functional diversification of FLOWERING LOCUS T/TERMINAL FLOWER 1 family genes in plants. Semin. Cell Dev. Biol. 109, 20–30. doi: 10.1016/j.semcdb.2020.05.007 32507412

[B20] Jin H. Tang X. Xing M. Zhu H. Sui J. Cai C.. (2019). Molecular and transcriptional characterization of phosphatidyl ethanolamine-binding proteins in wild peanuts Arachis duranensis and Arachis ipaensis. BMC Plant Biol. 19, 484. doi: 10.1186/s12870-019-2113-3 31706291 PMC6842551

[B21] Kaneko-Suzuki M. Kurihara-Ishikawa R. Okushita-Terakawa C. Kojima C. Nagano-Fujiwara M. Ohki I.. (2018). TFL1-like proteins in rice antagonize rice FT-like protein in inflorescence development by competition for complex formation with 14-3-3 and FD. Plant Cell Physiol. 59, 458–468. doi: 10.1093/pcp/pcy021 29401229

[B22] Kim G. Rim Y. Cho H. Hyun T. K. (2022). Identification and functional characterization of FLOWERING LOCUS T in Platycodon grandiflorus. Plants (Basel) 11, 325. doi: 10.3390/plants11030325 35161306 PMC8840131

[B23] Kobayashi Y. Kaya H. Goto K. Iwabuchi M. Araki T. (1999). A pair of related genes with antagonistic roles in mediating flowering signals. Science 286, 1960–1962. doi: 10.1126/science.286.5446.1960 10583960

[B24] Komeda Y. (2004). Genetic regulation of time to flower in Arabidopsis thaliana. Annu. Rev. Plant Biol. 55, 521–535. doi: 10.1146/annurev.arplant.55.031903.141644 15377230

[B25] Komiya R. Ikegami A. Tamaki S. Yokoi S. Shimamoto K. (2008). Hd3a and RFT1 are essential for flowering in rice. Development 135, 767–774. doi: 10.1242/dev.008631 18223202

[B26] Kotoda N. Hayashi H. Suzuki M. Igarashi M. Hatsuyama Y. Kidou S.. (2010). Molecular characterization of FLOWERING LOCUS T-like genes of apple (Malus x domestica Borkh.). Plant Cell Physiol. 51, 561–575. doi: 10.1093/pcp/pcq021 20189942

[B27] Kumar S. Stecher G. Tamura K. (2016). MEGA7: molecular evolutionary genetics analysis version 7.0 for bigger datasets. Mol. Biol. Evol. 33, 1870–1874. doi: 10.1093/molbev/msw054 27004904 PMC8210823

[B28] Lescot M. Déhais P. Thijs G. Marchal K. Moreau Y. Van de Peer Y.. (2002). PlantCARE, a database of plant cis-acting regulatory elements and a portal to tools for in silico analysis of promoter sequences. Nucleic Acids Res. 30, 325–327. doi: 10.1093/nar/30.1.325 11752327 PMC99092

[B29] Li H. Ran K. Dong Q. Zhao Q. Shi S. (2020). Cloning, sequencing, and expression analysis of 32 NAC transcription factors (MdNAC) in apple. PeerJ 8, e8249. doi: 10.7717/peerj.8249 32411503 PMC7210808

[B30] Liu J. Wang J. Wang M. Zhao J. Zheng Y. Zhang T.. (2021). Genome-wide analysis of the R2R3-MYB gene family in Fragaria × ananassa and its function identification during anthocyanins biosynthesis in pink-flowered strawberry. Front. Plant Sci. 12. doi: 10.3389/fpls.2021.702160 PMC8435842 34527006

[B31] Liu X. Zhao D. Ou C. Hao W. Zhao Z. Zhuang F. (2022). Genome-wide identification and characterization profile of phosphatidy ethanolamine-binding protein family genes in carrot. Front. Genet. 13. doi: 10.3389/fgene.2022.1047890 PMC9696379 36437940

[B32] Oda A. Narumi T. Li T. Kando T. Higuchi Y. Sumitomo K.. (2012). CsFTL3, a chrysanthemum FLOWERING LOCUS T-like gene, is a key regulator of photoperiodic flowering in chrysanthemums. J. Exp. Bot. 63, 1461–1477. doi: 10.1093/jxb/err387 22140240 PMC3276106

[B33] Périlleux C. Bouché F. Randoux M. Orman-Ligeza B. (2019). Turning meristems into fortresses. Trends Plant Sci. 24, 431–442. doi: 10.1016/j.tplants.2019.02.004 30853243

[B34] Rao X. Huang X. Zhou Z. Lin X. (2013). An improvement of the 2ˆ (-delta delta CT) method for quantitative real-time polymerase chain reaction data analysis. Biostat. Bioinform. Biomath. 3, 71–85. doi: 10.1089/cmb.2012.0279 PMC4280562 25558171

[B35] Remay A. Lalanne D. Thouroude T. Le Couviour F. Hibrand-Saint Oyant L. Foucher F. (2009). A survey of flowering genes reveals the role of gibberellins in floral control in rose. Theor. Appl. Genet. 119, 767–781. doi: 10.1007/s00122-009-1087-1 19533080

[B36] Song C. Li G. Dai J. Deng H. (2021). Genome-wide analysis of PEBP genes in Dendrobium huoshanense: unveiling the antagonistic functions of FT/TFL1 in flowering time. Front. Genet. 12. doi: 10.3389/fgene.2021.687689 PMC8299281 34306028

[B37] Song S. Wang G. Wu H. Fan X. Liang L. Zhao H.. (2020). OsMFT2 is involved in the regulation of ABA signaling-mediated seed germination through interacting with OsbZIP23/66/72 in rice. Plant J. 103, 532–546. doi: 10.1111/tpj.14748 32170894

[B38] Szklarczyk D. Gable A. L. Nastou K. C. Lyon D. Kirsch R. Pyysalo S.. (2021). The STRING database in 2021: customizable protein-protein networks, and functional characterization of user-uploaded gene/measurement sets. Nucleic Acids Res. 49, D605–D612. doi: 10.1093/nar/gkaa1074 33237311 PMC7779004

[B39] Takagi H. Hempton A. K. Imaizumi T. (2023). Photoperiodic flowering in Arabidopsis: Multilayered regulatory mechanisms of CONSTANS and the florigen FLOWERING LOCUS T. Plant Commun. 4, 100552. doi: 10.1016/j.xplc.2023.100552 36681863 PMC10203454

[B40] Venail J. da Silva Santos P. H. Manechini J. R. Alves L. C. Scarpari M. Falcão T.. (2022). Analysis of the PEBP gene family and identification of a novel FLOWERING LOCUS T orthologue in sugarcane. J. Exp. Bot. 5, 2035–2049. doi: 10.1093/jxb/erab539 PMC8982381 34893811

[B41] Wada K. C. Yamada M. Shiraya T. Takeno K. (2010). Salicylic acid and the flowering gene FLOWERING LOCUS T homolog are involved in poor-nutrition stress-induced flowering of Pharbitis nil. J. Plant Physiol. 167, 447–452. doi: 10.1016/j.jplph.2009.10.006 19906461

[B42] Wang Y. Tang H. Debarry J. D. Tan X. Li J. Wang X.. (2012). MCScanX: a toolkit for detection and evolutionary analysis of gene synteny and collinearity. Nucleic Acids Res. 40, e49. doi: 10.1093/nar/gkr1293 22217600 PMC3326336

[B43] Wang G. Wang P. Gao Y. Li Y. Wu L. Gao J.. (2018). Isolation and functional characterization of a novel FLOWERING LOCUS T homolog (NtFT5) in Nicotiana tabacum. J. Plant Physiol. 231, 393–401. doi: 10.1016/j.jplph.2018.10.021 30391867

[B44] Wen C. Zhao W. Liu W. Yang L. Wang Y. Liu X.. (2019). CsTFL1 inhibits determinate growth and terminal flower formation through interaction with CsNOT2a in cucumber. Development 146, dev180166. doi: 10.1242/dev.180166 31320327 PMC6679365

[B45] Wigge P. A. Kim M. C. Jaeger K. E. Busch W. Schmid M. Lohmann J. U.. (2005). Integration of spatial and temporal information during floral induction in Arabidopsis. Science 309, 1056–1059. doi: 10.1126/science.1114358 16099980

[B46] Wilkins M. R. Gasteiger E. Bairoch A. Sanchez J. C. Williams K. L. Appel R. D.. (1999). Protein identification and analysis tools in the ExPASy server. Methods Mol. Biol. 112, 531–552. doi: 10.1385/1-59259-584-7:531 10027275

[B47] Xi W. Yu H. (2010). MOTHER OF FT AND TFL1 regulates seed germination and fertility relevant to the brassinosteroid signaling pathway. Plant Signal Behav. 5, 1315–1317. doi: 10.4161/psb.5.10.13161 20935478 PMC3115377

[B48] Xu H. Guo X. Hao Y. Lu G. Li D. Lu J.. (2022). Genome-wide characterization of PEBP gene family in Perilla frutescens and PfFT1 promotes flowering time in Arabidopsis thaliana. Front. Plant Sci. 13. doi: 10.3389/fpls.2022.1026696 PMC9716100 36466292

[B49] Xu H. Yu Q. Shi Y. Hua X. Tang H. Yang L.. (2018). PGD: pineapple genomics database. Hortic. Res. 5, 66. doi: 10.1038/s41438-018-0078-2 30245835 PMC6139296

[B50] Yan X. Cao Q. Z. He H. B. Wang L. J. Jia. G. X. (2021). Functional analysis and expression patterns of members of the FLOWERING LOCUS T (FT) gene family in Lilium. Plant Physiol. Biochem. 163, 250–260. doi: 10.1016/j.plaphy.2021.03.056 33866146

[B51] Yue L. Li X. Fang C. Chen L. Yang H. Yang J.. (2021). FT5a interferes with the Dt1-AP1 feedback loop to control flowering time and shoot determinacy in soybean. J. Integr. Plant Biol. 63, 1004–1020. doi: 10.1111/jipb.13070 33458938

[B52] Zhang X. Henriques R. Lin S. S. Niu Q. W. Chua N. H. (2006). Agrobacterium-mediated transformation of Arabidopsis thaliana using the floral dip method. Nat. Protoc. 1, 641–646. doi: 10.1038/nprot.2006.97 17406292

[B53] Zhang Q. Li C. (2017). Comparisons of copy number, genomic structure, and conserved motifs for α-amylase genes from barley, rice, and wheat. Front. Plant Sci. 8. doi: 10.3389/fpls.2017.01727 PMC5633601 29051768

[B54] Zhang B. Li C. Li Y. Yu H. (2020). Mobile TERMINAL FLOWER1 determines seed size in Arabidopsis. Nat. Plants. 6, 1146–1157. doi: 10.1038/s41477-020-0749-5 32839516

[B55] Zhang H. Zhang Y. (2020). Molecularcloning and functional characterization of CmFT (FLOWERING LOCUS T) from Cucumis melo L. J. Genet. 99, 1–8. doi: 10.1007/s12041-020-1191-1 32529984

[B56] Zhu Y. Klasfeld S. Jeong C. W. Jin R. Goto K. Yamaguchi N.. (2020). TERMINAL FLOWER 1-FD complex target genes and competition with FLOWERING LOCUS T. Nat. Commun. 11, 5118. doi: 10.1038/s41467-020-18782-1 33046692 PMC7550357

